# Supplementary material for: Ingestion of Bifidobacterium longum changes miRNA levels in the brains of mice
Source: PLoS One. 2021 Apr 15;16(4):e0249817. doi: 10.1371/journal.pone.0249817 (PMC8049326; doi:10.1371/journal.pone.0249817)
Supplement: Attachment — Submitted filename: Response to Reviewers.docx [file pone.0249817.s003.docx]

Greetings,

We are thankful for the encouraging feedback from both reviewers. The more extensive recommendations from Reviewer 1 have certainly strengthened the paper. Every comment by both reviewers and the editor have been addressed. Specifically:

From the editor:

1. We have added the word, “E-mail” and the corresponding author’s initials to line 16 of the manuscript.
2. We added a caption and brief title for the Supporting Information to line 304 of the manuscript.
3. We added the ethics statement regarding animal work to the beginning of our Methods section. Lines 85 – 87.

From Reviewer 1:

1. We now reference an excellent review (4) that covers the role of the microbiota in neurodevelopment and behavior. Line 32 of the manuscript.
2. We have added additional references to support the claim regarding blood metabolites including a review of the field. Line 37 of the manuscript.
3. We have rewritten the sentence from lines 159 – 161 of the manuscript in order to clarify its meaning.
4. Our goal was to keep our conclusions modest, but we appreciate the encouragement to better highlight the importance of our results and how they connect to previous work. Multiple lines have been added to our General Conclusions section including a new important reference that gives further context for our work. We believe that these changes both strengthen the conclusions section and address this reviewers concern.

From Reviewer 2

1. This reviewer’s comments 1 and 3 regard the bowel microbiota which we did not measure. We now mention in the manuscript that we did not make this measurement. Line 103 of the manuscript.
2. We have added a reference (19) that supports our decision to follow up with mir-652. Line 192.

Thank you again to both reviewers for their thoughtful engagement with our paper.

Sincerely,

Dr. Clayton Carlson

Professor of Biology

Trinity Christian College

Palos Heights, Illinois, USA
